# The computationally designed TRI2-2 miniprotein inhibitor protects against multiple SARS-CoV-2 Omicron variants

**DOI:** 10.1038/s42003-025-09499-2

**Published:** 2026-01-10

**Authors:** Jimin Lee, James Brett Case, Young-Jun Park, Rashmi Ravichandran, Daniel Asarnow, M. Alejandra Tortorici, Jack T. Brown, Shilpa Sanapala, Lauren Carter, David Baker, Michael S. Diamond, David Veesler

**Affiliations:** 1 Department of Biochemistry, University of Washington, Seattle, WA USA; 2 Department of Medicine, Washington University School of Medicine, St. Louis, MO USA; 3 Howard Hughes Medical Institute, University of Washington, Seattle, WA USA; 4 Institute for Protein Design, University of Washington, Seattle, WA USA; 5 Department of Molecular Microbiology, Washington University School of Medicine, St. Louis, MO USA; 6 Department of Pathology and Immunology, Washington University School of Medicine, St. Louis, MO USA; 7 Andrew M. and Jane M. Bursky Center for Human Immunology and Immunotherapy Programs, Washington University School of Medicine, St. Louis, MO USA

**Keywords:** Cryoelectron microscopy, Protein design

## Abstract

The continued evolution of severe acute respiratory syndrome coronavirus 2 (SARS-CoV-2) has compromised neutralizing antibody responses elicited by prior infection or vaccination and abolished the utility of most monoclonal antibody therapeutics. We previously described a computationally-designed, homotrimeric miniprotein inhibitor, designated TRI2-2, that protects mice against pre-Omicron SARS-CoV-2 variants. Here, we show that TRI2-2 exhibits broadly neutralizing activity of SARS-CoV-2 variants and protects mice against BQ.1.1, XBB.1.5 and BA.2.86 challenge when administered intranasally post-exposure. The resistance of TRI2-2 to viral escape by most variants and the ability to deliver it directly to the upper airways highlight the potential of the multivalent miniprotein inhibitor as an alternative therapeutic modality.

## Abstract (web summary)

Computationally designed TRI2-2 miniprotein inhibitor broadly neutralizes multiple SARS-CoV-2 Omicron variants and confers post-exposure protection in mice against BQ.1.1, XBB.1.5, and BA.2.86 challenge when administered intranasally.

## Introduction

The severe acute respiratory syndrome coronavirus 2 (SARS-CoV-2) spike (S) glycoprotein interacts with its host receptor ACE2 and initiates viral entry into cells^1–4^. The emergence of SARS-CoV-2 Omicron variants at the end of 2021 and afterwards have reduced the efficacy of vaccines and monoclonal antibodies, increased the number of reinfections or breakthrough infections, and led to successive waves of global infection^5–10^.

We previously described a computationally-designed, homotrimeric miniprotein inhibitor, designated TRI2-2, that binds with high avidity to SARS-CoV-2 S as a result of simultaneously engaging all three receptor-binding domains (RBDs) within an S trimer^11,12^. We showed that intranasal administration of TRI2-2 after viral exposure protected mice from challenge with the SARS-CoV-2 Beta and Delta variants^12^. Here, we show that TRI2-2 retains in vitro neutralization and in vivo protective activity against virtually all Omicron variants that have emerged for 5 years of the COVID-19 pandemic, highlighting its robustness as a possible next-generation therapeutic.

## Results

**Fig. 1:**
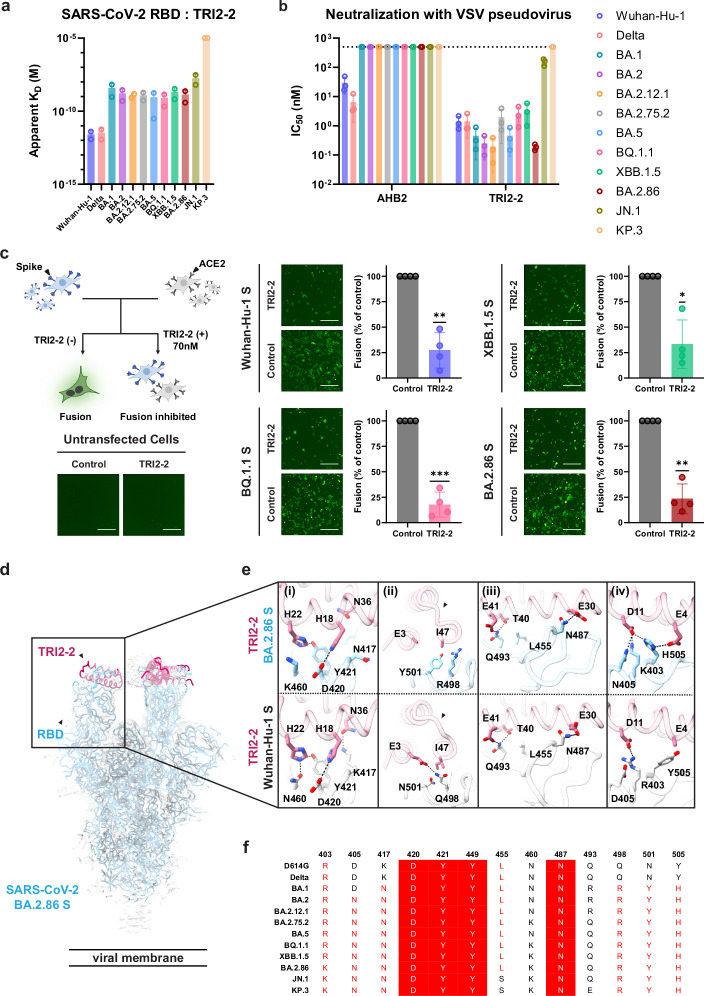
TRI2-2 cross-reacts with and potently neutralizes SARS-CoV-2 Omicron variants. **a** Binding of TRI2-2 to variant RBDs immobilized at the surface of BLI biosensors. Means of two biological replicates (each replicate is shown as a circle) are shown as bar graphs with lines representing SD. **b** Neutralization of SARS-CoV-2 S VSV pseudoviruses harboring the Wuhan-Hu-1 D614G, Delta, BA.1, BA.2, BA.2.12.1, BA.2.75.2, BA.5, BQ.1.1, XBB.1.5, BA.2.86, or JN.1 S. Means of three biological replicates (each replicate is shown as a circle) rendered as bar graphs with SD. **c** Cell-cell fusion assay between BHK21 cells expressing SARS-CoV-2 D614G, BQ.1.1, XBB.1.5, or BA.2.86 S glycoprotein and VeroE6-TMPRSS2 cells in the absence of TRI2-2 (control) or in the presence of 70 nM TRI2-2. Each dot represents replicate from four different biological replicates. SDs shown as lines. One sample *t* tests between control and TRI2-2 treatment; ns not significant; *p < 0.05, **p < 0.01, ***p < 0.001, ****p < 0.0001, *p* = 0.0035 (Wuhan-Hu-1), *p* = 0.0009 (BQ1.1), *p* = 0.0113 (XBB.1.5), *p* = 0.0018 (BA.2.86). The schematic of the cell-cell fusion assay was created in BioRender. Tortorici, A. (2026) https://BioRender.com/xjsmgd6. Scale bar: 1 mm. **d**, **e** CryoEM structure of TRI2-2 bound to the BA.2.86 S glycoprotein trimer (electron potential map shown as a semi-transparent gray surface) (**d**) and close-up views of the binding interface between TRI2-2 and BA.2.86 RBD compared to that obtained in complex with the Wuhan-Hu-1 RBD (PDB 7UHB). **e** Black arrows highlight the conformationally reorganized loop in (**e-ii**). **f** Amino acid sequences of the key residues at the binding interface. Coloring scheme follows ESPript 3^51^.

To investigate the ability of TRI2-2 to recognize SARS-CoV-2 Omicron variants associated with successive infection waves, we assessed binding to a panel of biotinylated RBDs immobilized on biolayer interferometry (BLI) biosensors. TRI2-2 bound to the Wuhan-Hu-1 and Delta RBDs with single digit picomolar avidities and to the BA.1, BA.2, BA.2.12.1, BA.2.75.2, BA.5, BQ.1.1, XBB.1.5, BA.2.86, and JN.1 variant RBDs with nanomolar avidities. However, no binding to the KP.3 RBD was detected (Figs. 1a and S1). These data establish that TRI2-2 binds avidly (i.e., with slow off-rates) to most Omicron variants evaluated despite accumulation of RBD mutations in the receptor-binding motif (ACE2-binding site), which overlaps with the TRI2-2-binding site.

We subsequently tested the ability of TRI2-2 to inhibit vesicular stomatitis virus (VSV) particles pseudotyped with the Wuhan-Hu-1 D614G, Delta, BA.1, BA.2, BA.2.12.1, BA.2.75.2, BA.5, BQ.1.1, XBB.1.5, BA.2.86, JN.1, or KP.3 S using HEK293T target cells stably expressing human ACE2^13^. TRI2-2 potently neutralized all pseudoviruses tested, in a concentration-dependent manner, with half-maximal inhibition concentrations ranging between ~0.5 and ~5 nM except for JN.1, for which its potency was reduced to ~300 nM and KP.3 that was not inhibited in our assay, concurring with its lack of binding to this variant RBD (Figs. 1b and S2). Comparatively, the AHB2 minibinder, which is the monomer from which TRI2-2 derives (through genetic fusion to a trimerization motif), inhibited Wuhan-Hu-1 D614G S- and Delta S VSV pseudoviruses but failed to block any of the Omicron variants evaluated (Figs. 1b and S2). These findings demonstrate that harnessing the binding avidity resulting from trivalent engagement of S trimers endows TRI2-2 with broadly neutralizing activity.

We subsequently assessed the ability of TRI2-2 to inhibit syncytia formation between cells using a split green fluorescent protein (GFP) system with Vero E6/TMPRSS2 target cells (Vero E6 cells stably expressing TMPRSS2 and GFP β strand 11) and BHK-21 effector cells (stably expressing GFP β strands 1 to 10) transiently transfected with Wuhan-Hu-1 D614G, BQ.1.1, XBB.1.5 or BA.2.86 S^6,14^. Consistent with the broadly neutralizing activity observed, addition of TRI2-2 decreased S-mediated syncytia formation with all variants tested (Figs. 1c and S3)^15^.

To determine the molecular basis of the exceptional resilience to antigenic changes that have emerged in the S glycoprotein of SARS-CoV-2 variants, we determined a cryoEM structure of the BA.2.86 S trimer bound to TRI2-2 at 2.4 Å resolution (Figs. 1d and S4). Local refinement of the region comprising the RBD and TRI2-2 yielded a reconstruction at 3.2 Å resolution with improved local resolution, revealing key interacting residues that are mutated or conserved in the SARS-CoV-2 Wuhan-Hu-1 RBD and BA.2.86 RBD (Fig. 1e). The BA.2.86 S K417N_SARS-CoV-2_ (Fig. 1e-i) and Q498R_SARS-CoV-2_ (Fig. 1e-ii) mutations abolish hydrogen bonds formed with N36_TRI2-2_ and E3_TRI2-2_, respectively, possibly contributing to the reduced binding avidity observed for Omicron variants relative to SARS-CoV-2 Wuhan-Hu-1 and Delta. The Q498R and N501Y mutations are sterically incompatible with the positioning of the TRI2-2 I47 side chain observed in the Wuhan-Hu-1 complex structure and leads to reorganization of the minibinder region comprising the C-terminal part of the second helix and loop connecting to the third helix (Fig. 1e-ii, highlighted with black arrows). Our structure also suggests that the JN.1 L455S_SARS-CoV-2_ residue substitution would reduce van der Waals packing at the interface with the minibinder (Fig. 1e-iii), thereby decreasing TRI2-2 binding avidity and consequently neutralizing activity (Fig. 1a, b). However, hydrogen bonds formed between H18_TRI2-2_ and D420_SARS-CoV-2_ (Fig. 1e-i), H22_TRI2-2_ and Y421_SARS-CoV-2_ (Fig. 1e-i), E30_TRI2-2_ and N487_SARS-CoV-2_ (Fig. 1e-iii), T40_TRI2-2_/E41_TRI2-2_ and Q493_SARS-CoV-2_ (Fig. 1e-iii), are conserved in the Wuhan-Hu-1 and BA.2.86 RBD complex structures. We note that although BA.1 and BA.2 harbored the Q493R substitution^6,7^, TRI2-2 retained potent neutralizing activity against these variants (Fig. 1b, f). However, the KP.3 Q493E_SARS-CoV-2_ mutation (Fig. 1e-iii) would introduce charge repulsion with the nearby E41_TRI2-2_ residue (Fig. 1e-iii), which likely explains the loss of binding and of neutralizing activity of this variant. Contacts in several regions are remodeled by residue changes between Wuhan-Hu-1 and BA.2.86, as revealed by our structural data. The BA.2.86 S Y505H substitution leads to formation of a salt bridge triad with TRI2-2 residues E4 and D11, whereas D11 is salt bridged and hydrogen bonded to K403 and N405 (which are mutated from R403 and D405 in Wuhan-Hu-1 S), respectively (Fig. 1e-iv). Moreover, the BA.2.86 S N460K_SARS-CoV-2_ mutation replaces the hydrogen bond formed with H22_TRI2-2_ by a cation-pi interaction with the same residue (Fig. 1e-i). Overall, TRI2-2 buries a comparable surface at the interface with the Wuhan-Hu-1 and the BA.2.86 RBDs despite the aforementioned residue mutations. These data explain the retained TRI2-2 binding and neutralization of virtually all SARS-CoV-2 variants tested despite variations within the targeted epitope that are accommodated.

**Fig. 2:**
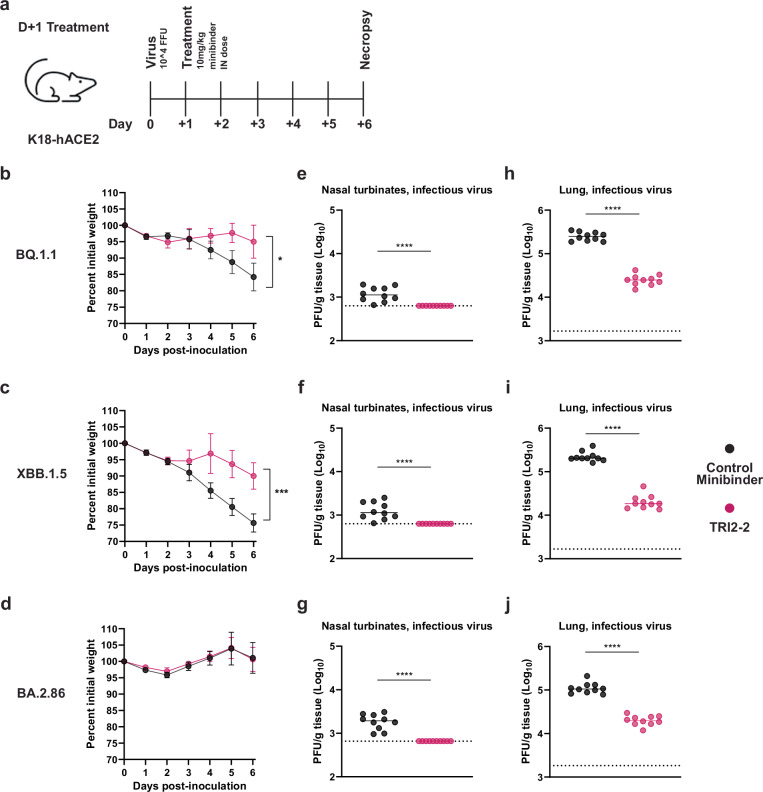
Post-exposure TRI2-2 administration protects mice challenged with the SARS-CoV-2 BQ.1.1, XBB.1.5, and BA.2.86 variants. **a** Schematic of study design. **b–d** Weight loss for mice challenged with BQ.1.1 (**b**), XBB.1.5 (**c**), or BA.2.86 (**d**) (dots represent mean and lines represent SD.; *n* = 10 mice per group per challenge virus; differences in area under the curves assessed by Student’s *t*-test with Welch’s correction for each virus; *P < 0.01; ***P < 0.001). **e**–**g** Nasal turbinate infectious viral titers for mice challenged with BQ.1.1 (**e**), XBB.1.5 (**f**), or BA.2.86 (**g**). **h**–**j** Lung infectious viral titers for mice challenged with BQ.1.1 (**h**), XBB.1.5 (**i**), or BA.2.86 (**j**) (solid lines indicate median values; dotted lines indicate limit of detection of assay, *n* = 10 mice per group per challenge virus, two experiments; Two-tailed Mann–Whitney test between control and TRI2-2 treatment; ****p < 0.0001 (**e**–**j**).

To study the protective efficacy of TRI2-2 in vivo against immune evasive SARS-CoV-2 Omicron variants, we intranasally inoculated highly susceptible K18-hACE2 mice^16^ with 10^4^ FFU of BQ.1.1, XBB.1.5, or BA.2.86. One day later, we intranasally administered a single 10 mg/kg dose of TRI2-2 or an influenza virus control minibinder^17^. For all variants evaluated, post-exposure TRI2-2 treatment protected against weight loss throughout the duration of the experiments and reduced viral titers in the lungs and nasal turbinates six days post-challenge as compared to the control minibinder (Figs. 2 and S5). These results indicate that intranasal administration of TRI2-2 confers protection against SARS-CoV-2 challenge in a stringent model of disease with three key SARS-CoV-2 Omicron variants.

## Discussion

Intramuscular vaccination results in lower neutralizing antibody titers within the human upper respiratory tract compared to natural infection^18–20^. These findings, coupled with waning natural immunity and viral antigenic changes, likely contribute to susceptibility to SARS-CoV-2 breakthrough infections. This results in the continued transmission of SARS-CoV-2 globally, and motivates the development of next-generation countermeasures that may be administered intranasally or orally. Preclinical assessment of intranasally administered influenza and sarbecovirus vaccine candidates demonstrated the induction of lung-resident protective mucosal humoral and cellular immunity at the site of viral entry^21–25^ and lipopeptide fusion inhibitors prevented SARS-CoV-2 direct-contact transmission in ferrets^26^. Furthermore, post-exposure prophylaxis nasal spray administration of the SA58 monoclonal antibody in humans was shown to markedly reduce the risk of contracting COVID-19^27^.

The computationally-designed TRI2-2 minibinder mediates broadly neutralizing activity and in vivo protection of mice in both the upper and lower airways against the highly immune evasive SARS-CoV-2 BQ.1.1, XBB.1.5, and BA.2.86 variants. These data show that TRI2-2 can accommodate residue substitutions within its epitope and provide a molecular framework to explain the remarkable neutralization breadth of SARS-CoV-2 variants that have emerged since the start of the pandemic more than five years ago, with the exception of KP.3 due to the Q493E residue mutation. TRI2-2 is therefore endowed with exceptionally broad neutralizing activity, outperforming all monoclonal antibodies developed against SARS-CoV-2 except VIR-7229^28^ and SA55^29^. Moreover, TRI2-2 has exceptional biophysical stability, enabling cost-effective, large-scale production, setting it apart from monoclonal antibodies that are expensive to manufacture and more challenging to scale.

Recent advances in computational protein design have markedly accelerated the workflow enabling design and optimization of minibinders^30–33^. Additionally, our cryo-EM structure of the BA.2.86 RBD bound to TRI2-2 will guide the future design of TRI2-2 derivative minibinders to accommodate new mutations that abrogate the binding between TRI2-2 and the SARS-CoV-2 KP.3 variant. The low manufacturing cost and the rapid design workflow along with antiviral efficacy when delivered to the upper respiratory tract are attractive properties of minibinders that could change the drug development paradigm against rapidly evolving respiratory viruses of public health concern.

## Methods

### Cells

Cell lines used in this study were DH10B competent cells (Thermo Fisher Scientific), HEK293T (ATCC, CRL-11268), Vero E6-TMPRSS2-GFP_11_, and BHK-21-GFP_1–10_^6^ and HEK293T cells with stable human ACE2 expression (kindly provided by Jesse Bloom)^13^. Cells were cultured in 10% fetal bovine serum (FBS) (Fisher Scientific-Cytiva), 1% penicillin-streptomycin (Thermo Fisher Scientific) Dulbecco’s modified Eagle medium (DMEM) at 37 °C, 5% CO_2_. BHK-21-GFP_1–10_ and Vero E6-TMPRSS2-GFP_11_ cells were generated in-house and were cultured supplemented with 2 µg/mL of Puromycin for BHK and 8 µg/mL of Puromycin and 4 µg/mL of Blasticidin for Vero cells in 10% FBS (Fisher Scientific-Cytiva), 1% penicillin-streptomycin (Thermo Fisher Scientific) DMEM at 37 °C, 5% CO_2_. Expi293F (Thermo Fisher Scientific) were cultured at 37 °C and 8% CO_2_. None of the cell lines were authenticated or tested for mycoplasma contamination.

Vero-TMPRSS2^34^ cells were cultured at 37 °C in DMEM supplemented with 10% FBS, 10 mM HEPES pH 7.3, 1 mM sodium pyruvate, 1× non-essential-essential amino acids, and 100 U/mL of penicillin-streptomycin. Vero-TMPRSS2 cells were supplemented with 5 µg/mL of blasticidin. Vero-hACE2-TMPRSS2 cells were supplemented with 10 µg/mL of puromycin. All cells routinely tested negative for mycoplasma using a PCR-based assay.

### Production of recombinant SARS-CoV-2 S RBDs

The SARS-CoV-2 RBDs were expressed in Expi293F cells (Thermo) at 37 °C and 8% CO_2_. Cells were transfected with the corresponding plasmids using Expifectamine (Thermo) following the manufacturer’s protocol. Four days post-transfection, supernatants were clarified by centrifugation at 3000 × *g* for 30 min, supplemented with 25 mM phosphate pH 8.0, and 300 mM NaCl and then passed through a 0.22 µm sterile filter. Supernatants were then bound to 1 mL Histrap Excel columns (Cytiva) previously equilibrated in 25 mM phosphate pH 8.0, 300 mM NaCl. Nickel columns were washed with 25 mM phosphate pH 8.0, 300 mM NaCl, and 10 mM imidazole prior to elution with 25 mM phosphate pH 8.0, 300 mM NaCl, and 300 mM imidazole. After buffer exchanging into 20 mM phosphate pH 8.0 and 100 mM NaCl using a centrifugal filter device with a MWCO of 10 kDa, the purified RBDs were biotinylated using the BirA biotin-protein ligase reaction kit (Avidity). The biotinylated RBD’s were bound, washed, and eluted again on the same affinity column. Purified biotinylated RBD’s were then concentrated and eluted on a Superdex200 increase 10/300 size-exclusion column (Cytiva) equilibrated in 20 mM phosphate pH 8.0 and 100 mM NaCl. Fractions containing monomeric and monodisperse RBDs were flash frozen and stored at −80 °C until use.

### Production of recombinant TRI2-2 and influenza virus minibinders

The TRI2-2 and influenza virus minibinders were cloned into pET29b between the NdeI and XhoI restriction sites by Genscript. The TRI2-2 minibinder was cloned with a C-terminal polyhistidine tag and the influenza minibinder was cloned with an N-terminal polyhistidine tag (MHHHHHHGSDDDSHKKKLEDELESLIKKARNPEARKMVEWLARKAKKAADLGNKISVEFLLRLARQFAEASAR)^17^. Minibinders were expressed in Lemo21(DE3) cells (NEB) in TB II Media (MP Bio) at 37 °C with IPTG induction. After cell harvest, pellets were resuspended in Gibco dPBS and lysed by microfluidization at 18,000 psi. Whole cell lysates were clarified by centrifugation at 18,000 × *g* for 30 min and supernatants were then bound to a 5 mL Histrap Nickel Sepharose FF column (Cytiva) previously equilibrated in Gibco dPBS supplemented with 30 mM Imidazole. Nickel columns were washed with Gibco dPBS (ThermoFisher) supplemented with 30 mM imidazole prior to elution with Gibco dPBS supplemented with 500 mM Imidazole. Using a centrifugal filter device with a MWCO of 3 kDa, the IMAC fractions containing minibinders of interest were concentrated and then further purified by size-exclusion chromatography using an Superdex S75 Increase 10/300 GL column (Cytiva) equilibrated in Gibco dPBS as running buffer. Fractions containing TRI2-2 or Influenza minibinders were concentrated (as needed), filtered with a 0.2 µm filter, and then tested for endotoxin (LAL Charles River) prior to being flash frozen and stored at −80 °C until use.

### Binding analysis using biolayer interferometry (BLI)

BLI binding assays were performed on an Octet Red (Sartorius) instrument operated at 30 °C with shaking (1000 rpm). Streptavidin biosensors were hydrated in a 10× kinetics buffer (Sartorius) for 10 min prior to the experiment. Biosensors were incubated in a 10× kinetics buffer for 60 s followed by the loading of biotinylated RBDs to the tip, all to a final level of 1 nm. Loaded biosensors were equilibrated in a 10× kinetics buffer for 120 s. For affinity binding assays to determine K_D_ values, RBD-loaded tips were dipped into a concentration series of TRI2-2 (threefold serial dilution from 25 nM to 0.9 nM) for 300 s followed by 300 s of dissociation in a 10× kinetics buffer. Global fits were used to calculate K_D_ values using a 1:1 binding fit model. Data were plotted using GraphPad Prism. Assays were replicated with two biological replicates (recombinant RBD proteins generated on different days) and representative graphs and values are shown in Supplementary Fig. 1 and Supplementary Table 1, respectively.

### Production of VSV pseudoviruses

SARS-CoV-2 spike VSV pseudoviruses were produced using HEK293T cells seeded on BioCoat Cell Culture Dish: poly-D-Lysine 100 mm (Corning). The following day, cells were transfected with spike constructs using Lipofectamine 2000 (Thermo Fisher Scientific) in Opti-MEM transfection medium. After 5 h of incubation at 37 °C with 5% CO2, cells were supplemented with DMEM containing 10% of FBS. On the next day, cells were washed with three times with DMEM and infected with VSV (G*ΔG-luciferase) for 2 h, followed by five washes with DMEM medium before addition of anti-VSV G antibody (I1-mouse hybridoma supernatant diluted 1:40, ATCC CRL-2700) and medium. After 18–24 h of incubation at 37 °C with 5% CO_2_, pseudoviruses were collected and cell debris removed by centrifugation at 3000 × *g* for 10 min. Pseudoviruses were further filtered using a 0.45 µm syringe filter and concentrated 10× prior to storage at −80 °C.

### Neutralization assays

For SARS-CoV-2 S VSV neutralization with TRI2-2 and AHB2, HEK293T cells with stable human ACE2 expression in DMEM supplemented with 10% FBS and 1% PenStrep were seeded at 40,000 cells/well into 96-well plates [3610] (Corning) coated with poly-lysine [P4707] (Sigma) and incubated overnight at 37 °C. The following day, a half-area 96-well plate (Greiner) was prepared with threefold serial dilutions of TRI2-2 and AHB2 with a starting concentration of 1 μM. An equal volume of DMEM with diluted pseudoviruses was added to each well. All pseudoviruses were diluted between 1:3 and 1:27 to reach a target entry of ~10^6^ RLU. The mixture was incubated at room temperature for 45–60 min. Media was removed from the cells, and 40 μL from each well of the half-area 96-well plate containing minibinder and pseudovirus were transferred to the 96-well plate seeded with cells and incubated at 37 °C for 1 h. After 1 h, an additional 40 μL of DMEM supplemented with 20% FBS and 2% PenStrep was added to the cells. After 18–20 h, 40 μL of One-Glo-EX substrate (Promega) was added to each well and incubated on a plate shaker in the dark for 5 min before reading the relative luciferase units using a BioTek Neo2 plate reader. Relative luciferase units were plotted and normalized in Prism (GraphPad): 0% entry being cells lacking pseudovirus and 100% entry being cells containing virus but lacking minibinder. Prism (GraphPad) nonlinear regression with “[Inhibitor] versus normalized response with a variable slope” was used to determine IC_50_ values from curve fits with 3 technical repeats. Three biological replicates were carried out for each sample-pseudovirus pair.

### Fusion assays

Cell-to-cell fusion assay using a split-GFP system was conducted as previously described. BHK-21-GFP_1–10_ cells were split into 6-well plates at a density of 250,000 cells per well. The following day, the growth medium was removed from the 6-well plates and cells were washed with DMEM followed by addition of the growth medium. Then, the cells were transfected with 4 µg of S protein DNA using Lipofectamine 2000 transfection kit. Vero E6-TMPRSS2-GFP_11_ cells were plated into 96-well, glass bottom, black-walled plates (CellVis) at a density of 36,000 cells per well. Twenty-four hours after transfection, BHK-21-GFP_1–10_ cells expressing the S protein were washed three times using FluoroBrite DMEM (Thermo Fisher) and detached using an enzyme-free cell dissociation buffer (Gibco). 9000 BHK-21-GFP_1–10_ cells were added to each well with or without TRI2-2 with the 1:4 serial dilution starting from the initial concentration of 70 nM, and the mixture was incubated at 37 °C and 5% CO_2_ for 2 h before being transferred on top of the Vero E6-TMPRSS2-GFP_11_ that was washed three times with FluoroBrite DMEM. The mixture was incubated at 37 °C and 5% CO_2_ in a Cytation 7 plate Imager (BioTek) and both bright-field and GFP images were collected every 30 min for 18 h. Fusogenicity was assessed by measuring the area showing GFP fluorescence for each image using Gen5 Image Prime v3.11 software. Raw grayscale 16-bit images were pseudocolored in ImageJ using Green Hot Look Up Table.

### Production of recombinant SARS-CoV-2 S BA.2.86

The SARS-CoV-2 BA.2.86 hexapro S ectodomain construct includes its native signal peptide, hexapro mutations (F817P, A892P, A899, A942P, K986P, V987P), and a C-terminal foldon, avi tag, and a 8× histidine tag. SARS-CoV-2 BA.86 hexapro S ectodomain was expressed in Expi293F cells (Thermo) at 37 °C and 8% CO_2_. Cells were transfected using Expifectamine293 (Thermo) following the manufacturer’s protocol. Four days post-transfection, Expi293F cell supernatant was clarified by centrifugation at 4121 × *g* for 30 min, supplemented with 25 mM phosphate pH 8.0, 300 mM NaCl. Supernatant was then bound to His-Trap Excel column (Cytiva) previously equilibrated in 25 mM phosphate pH 8.0, 300 mM NaCl. Nickel columns were washed with 20–40 mL of 25 mM phosphate pH 8.0, 300 mM NaCl, and 40 mM Imidazole. S protein was eluted using 25 mM phosphate pH 8.0, 300 mM NaCl, and 300 mM imidazole prior to being buffer exchanged to 50 mM Tris-HCl pH 8.0, 150 mM NaCl using a centrifugal filter device with a MWCO of 100 kDa. Protein was then flash frozen and stored at −80 °C.

### Cryo-EM sample preparation and data collection

The SARS-CoV-2 BA.2.86 S complex with TRI2-2 at a molar ratio of 1:8 just before the grid preparation. The cryo-EM dataset was collected over two different sessions, which were combined to be processed together. 3 µL of SARS-CoV-2 BA.2.86 S (Acro Biosystems, SPN-C524y) complex with TRI2-2 at 0.6 mg/mL was added to a glow discharged (30 s at 15 mA) UltraAuFoil R1.2/1.3:Au300 grid^35^ prior to plunge freezing using a vitrobot MarkIV (ThermoFisher Scientific) with a blot force of -1, wait time of 10 s, and 6 s blot time at 100% humidity and 22 °C. 3.5 µL of SARS-CoV-2 BA.2.86 S produced following the aforementioned protein production complex with TRI2-2 at 0.2 mg/mL was added to a glow discharged (10 s at 15 mA) Quantifoil 2 nm C Au300 grid prior to plunge freezing using a vitrobot MarkIV (ThermoFisher Scientific) with a blot force of −1, 4 s blot time, and 10 s wait time at 100% humidity and 22 °C. Data were acquired using an FEI Titan Krios transmission electron microscope operated at 300 kV and equipped with a Gatan K3 direct detector and Gatan Quantum GIF energy filter, operated in zero-loss mode with a slit width of 20 eV. Automated data collection was carried out using serialEM^36^ at a nominal magnification of 105,000× with a pixel size of 0.829 Å. The dose rate was adjusted to 53e^−^/Å^2^, and each movie was acquired in counting mode fractionated in 79 frames of 50 ms for UltraAuFoil and 99 frames of 40 ms for Quantifoil dataset, respectively. A total of 20,217 and 15,703 micrographs were collected for each datasets, respectively. Stage was tilted 0, 30, and 45 degrees for collection with the UltraAuFoil grid.

### Cryo-EM data processing, model building, and refinement

Particles were extracted with a box size of 320 pixels with a pixel size of 1.658 Å using WARP. Two rounds of reference-free 2D classification were performed using CryoSPARC^37^ to select well-defined particle images from each dataset. Particles belonging to classes with the best resolved spike protein density were selected. To improve particle picking further, we trained the Topaz^38^ picker on Warp-picked particle sets belonging to the selected classes after 2D classification. The particles picked using Topaz were extracted and subjected to 2D classification using cryoSPARC. The two different particle sets picked from Warp and Topaz were merged and duplicate particle picks were removed using a minimum distance cutoff of 90 Å. Initial model generation was done using ab-initio reconstruction in cryoSPARC and used as references for a heterogenous 3D refinement in cryoSPARC. After two rounds of ab-initio reconstructions and heterogeneous refinements to remove junk particles, 3D refinement was carried out using non-uniform refinement in cryoSPARC^39^ and the particles were transferred from cryoSPARC to Relion using pyem^40^ (https://github.com/asarnow/pyem) to be subjected to the Bayesian polishing procedure implemented in Relion^41^ during which particles were re-extracted with a box size of 512 pixels and a pixel size of 1.0 Å. After ab-initio reconstructions and heterogeneous refinements to select best class, subsequent 3D refinement used non-uniform refinement along with per-particle defocus refinement in cryoSPARC to yield the final reconstruction at 2.4 Å resolution comprising 811,069 particles. To further improve the density at the RBD:TRI2-2 interface, 3D classification and local refinement was performed using Relion and cryoSPARC with a soft mask comprising the RBD and TRI2-2, yielding a reconstruction at 3.2 Å resolution enabling model building. Reported resolutions are based on the 0.143 gold-standard Fourier shell correlation (FSC) criterion, and FSC curves were corrected for the effects of soft masking by high-resolution noise substitution^42,43^. To further improve the N-terminus domain (NTD) density, particles belonging to the best selected classes were subjected to another round of heterogeneous refinement, followed by non-uniform refinement with per-particle defocus. Particles were then symmetry expanded following the C3 axis and local refinement was performed using cryoSPARC with a soft mask comprising the NTD domain, yielding a reconstruction at 2.8 Å resolution, enabling model building. UCSF Chimera, UCSF ChimeraX, ModelAngelo^44^, and Coot were used to fit and rebuild atomic models into the cryoEM maps utilizing sharpened and unsharpened maps. The models were refined and relaxed using Rosetta^45,46^ and Phenix^47^, and validated using Phenix^47^, Molprobity^48^, and Privateer^49^.

### Virus

The BQ.1.1 (hCoV-19/USA/CA-Stanford-106_S04/2022; EPI_ISL_15196219) and XBB.1.5 hCoV-19/USA/MD-HP40900-PIDYSWHNUB/2022; EPI_ISL_16026423 strains were obtained from nasopharyngeal isolates and provided as generous gifts by Mehul Suthar (Emory University) and Andrew Pekosz (Johns Hopkins), respectively. All virus stocks were generated in Vero-TMPRSS2 cells and subjected to next-generation sequencing as described previously^34^ to confirm the presence and stability of expected substitutions. All virus experiments were performed in an approved biosafety level 3 facility.

### Mouse experiments

Animal studies were carried out in accordance with the recommendations in the Guide for the Care and Use of Laboratory Animals of the National Institutes of Health. The protocols were approved by the Institutional Animal Care and Use Committee at the Washington University School of Medicine (assurance number A3381-01). Virus inoculations were performed under anesthesia that was induced and maintained with ketamine hydrochloride and xylazine, and all efforts were made to minimize animal suffering. We have complied with all relevant ethical regulations for animal use.

Heterozygous K18-hACE2 C57BL/6J mice (strain: 2B6.Cg-Tg(K18-ACE2)2Prlmn/J) were obtained from The Jackson Laboratory. All animals were housed in groups and fed standard chow diets. For all mouse experiments, 8-week-old female K18-hACE2 mice were administered 10^4^ FFU of the respective SARS-CoV-2 strains by intranasal administration. One day later, animals were administered a single 10 mg/kg dose of influenza-specific control or TRI2-2 minibinder intranasally. All animals were monitored for body weight loss until being humanely euthanized at the indicated time post-infection. In vivo studies were not blinded, and mice were randomly assigned to treatment groups. No sample-size calculations were performed to power each study. Instead, sample sizes were determined based on prior in vivo virus challenge experiments.

### Measurement of viral RNA levels

Tissues were weighed and homogenized with zirconia beads in a MagNA Lyser instrument (Roche Life Science) in 1 mL of DMEM medium supplemented with 2% heat-inactivated FBS. Tissue homogenates were clarified by centrifugation at 10,000 rpm for 5 min and stored at −80 °C. RNA was extracted using the MagMax mirVana Total RNA isolation kit (Thermo Fisher Scientific) on the Kingfisher Flex extraction robot (Thermo Fisher Scientific). RNA was reverse transcribed and amplified using the TaqMan RNA-to-CT 1-Step Kit (Thermo Fisher Scientific). Reverse transcription was carried out at 48 °C for 15 min followed by 2 min at 95 °C. Amplification was accomplished over 50 cycles as follows: 95 °C for 15 s and 60 °C for 1 min. Copies of SARS-CoV-2 N gene RNA in samples were determined using a previously published assay^50^. Briefly, a TaqMan assay was designed to target a highly conserved region of the N gene (Forward primer: ATGCTGCAATCGTGCTACAA; Reverse primer: GACTGCCGCCTCTGCTC; Probe: /56-FAM/TCAAGGAAC/ZEN/AACATTGCCAA/3IABkFQ/). This region was included in an RNA standard to allow for copy number determination down to 10 copies per reaction. The reaction mixture contained final concentrations of primers and probes of 500 and 100 nM, respectively.

### Viral plaque assay

Vero-TMPRSS2-hACE2 cells were seeded at a density of 1 × 10^5^ cells per well in 24-well tissue culture plates. The following day, medium was removed and replaced with 200 µL of material to be titrated, diluted serially in DMEM supplemented with 2% FBS. After 1 h, 1 mL of methylcellulose overlay was added. Plates were incubated for 72 h, then fixed with 4% paraformaldehyde (final concentration) in phosphate-buffered saline (PBS) for 20 min. Plates were stained with 0.05% (wt/vol) crystal violet in 20% methanol and washed twice with distilled, deionized water.

### Statistics and reproducibility

All statistical tests were performed as described in the indicated figure legends using Prism 9.4.1 or 10.1.1. When comparing against control value in fusion assay, one sample *t* test was performed to determine statistical significance. When comparing two groups in viral challenge studies, a Mann–Whitney test was performed to determine statistical significance. The number of independent experiments performed is indicated in the relevant figure legends.

### Reporting summary

Further information on research design is available in the Nature Portfolio Reporting Summary linked to this article.

## Supplementary information

Supplementary_information

Description of Additional Supplementary Files

Supplementary_Data_1

Reporting Summary

## Data Availability

The sharpened and unsharpened cryoEM reconstructions and atomic models of SARS-CoV-2 BA.2.86 S in complex with TRI2-2 minibinder, SARS-CoV-2 BA.2.86 RBD in complex with TRI2-2 minibinder, and SARS-CoV-2 BA.2.86 NTD have been deposited in the Electron Microscopy Data Bank and the Protein Data Bank with accession codes EMD-45972 and PDB 9CWR (SARS-CoV-2 BA.2.86 S in complex with TRI2-2 minibinder), EMD-45969 and PDB 9CWP (SARS-CoV-2 BA.2.86 RBD in complex with TRI2-2 minibinder), and EMD-45971 and PDB 9CWQ (SARS-CoV-2 BA.2.86 NTD). The source data underlying the figures can be found in the Supplementary Data 1 file. Other data will be available from the corresponding author upon request.
