# Supplementary material for: The computationally designed TRI2-2 miniprotein inhibitor protects against multiple SARS-CoV-2 Omicron variants
Source: Commun Biol. 2026 Jan 10;9:224. doi: 10.1038/s42003-025-09499-2 (PMC12901202; doi:10.1038/s42003-025-09499-2)
Supplement: Supplementary file 1 — Supplementary_information [file 42003_2025_9499_MOESM1_ESM.pdf]

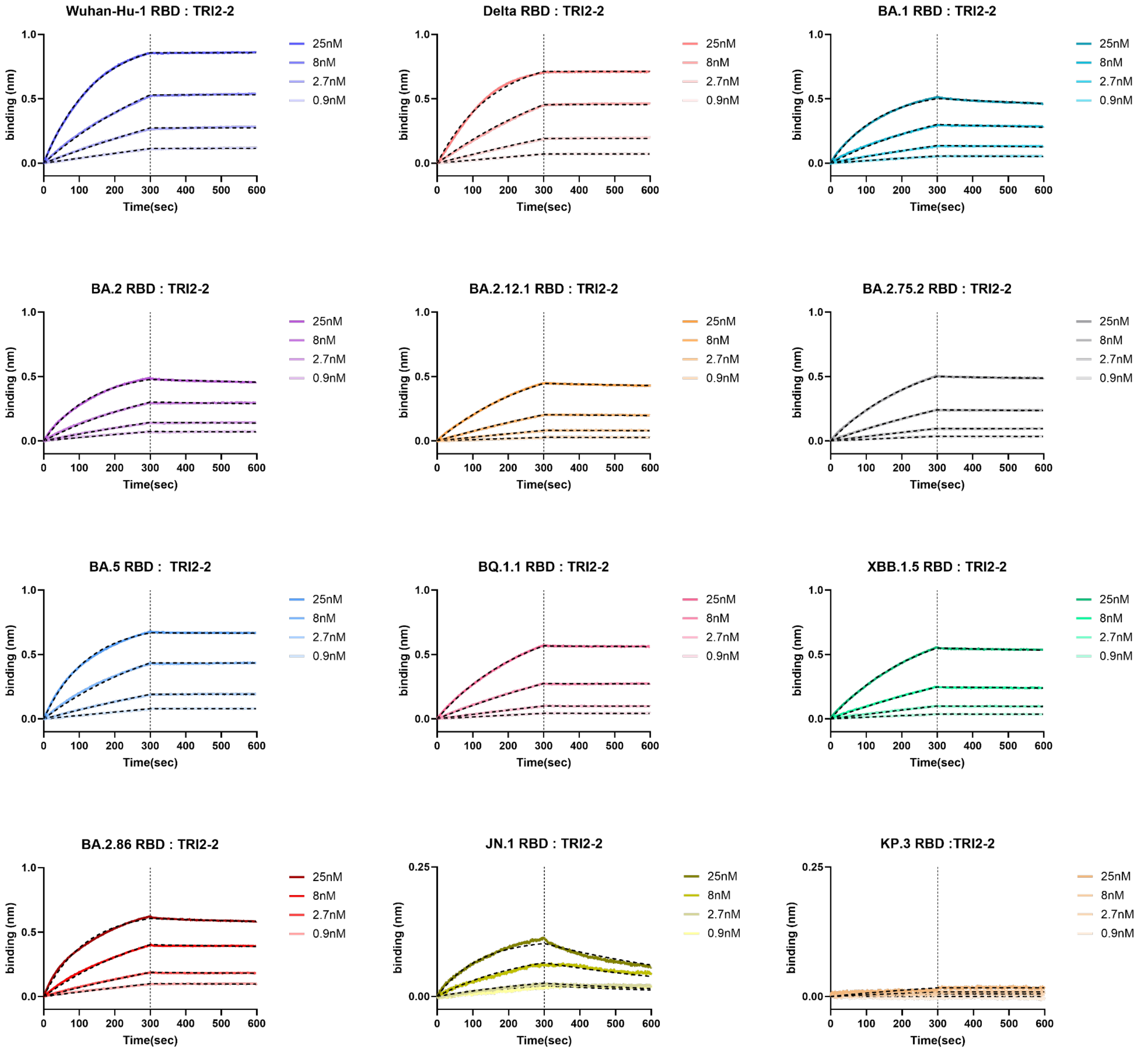

Supplementary Figure 1. Kinetic analysis of TRI2-2 binding to SARS-CoV-2 variant RBDs using biolayer interferometry. Biotinylated RBDs were immobilized on streptavidin biosensors to a final level of 1 nm shift each. The TRI2-2 concentrations used are provided in the color keys. Dashed black lines represent curve fits obtained using global fitting and a 1:1 binding model in the ForteBio BLI software. Representative graphs are shown from two biological replicates.

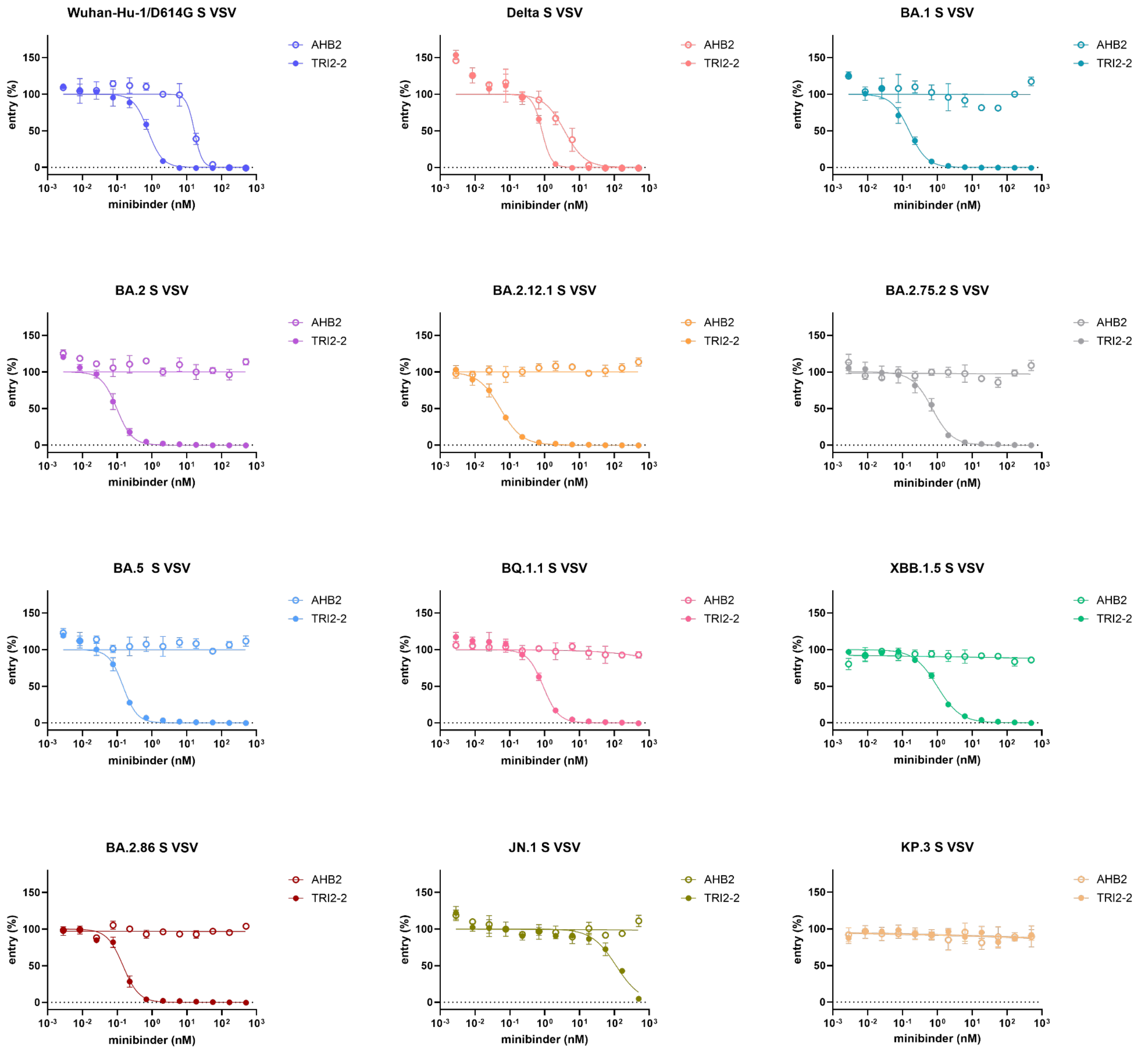

Supplementary Figure 2. Dose-response curves for neutralization of SARS-CoV-2 S variant VSV pseudoviruses by the TRI2-2 and AHB2 miniprotein inhibitors. Each dot represents the mean of three technical replicates. SD shown as lines. Representative graphs are shown from three biological replicates.

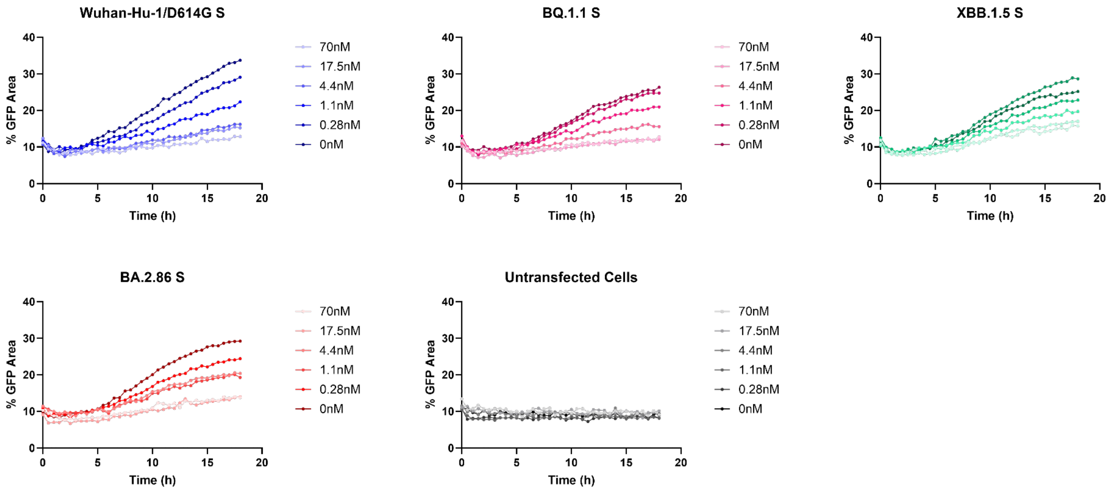

Supplementary Figure 3. Dose-response curves for TRI2-2-mediated fusion inhibition of SARS-CoV-2 S variants. Representative graphs are shown from four biological replicates.

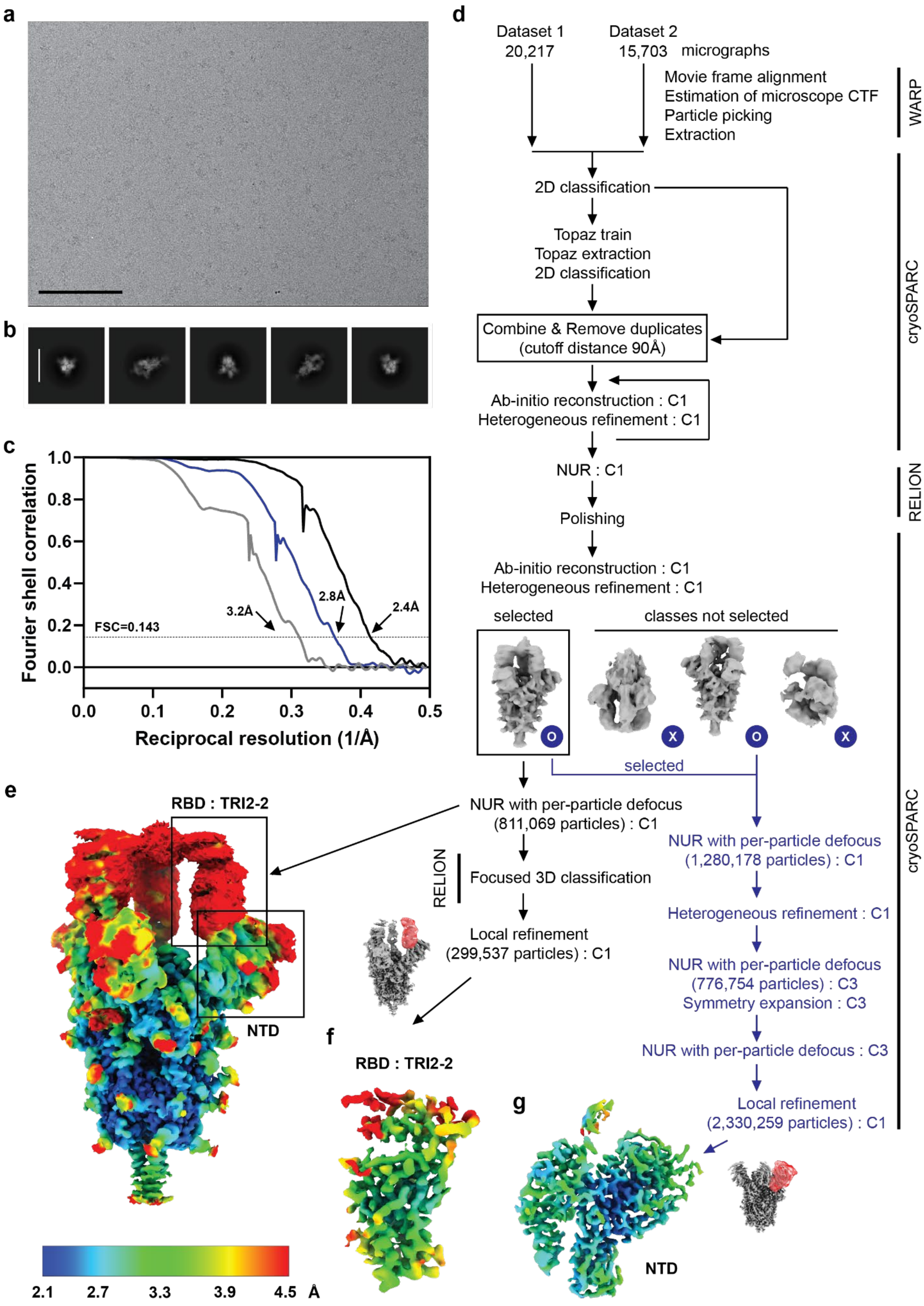
a
b
c
1.0
0.8
0.6
0.4
0.2
0.0
Fourier shell correlation
FSC=0.143
3.2Å
2.8Å
2.4Å
0.0
0.1
0.2
0.3
0.4
0.5
Reciprocal resolution (1/Å)
d
Dataset 1
20,217
Dataset 2
15,703
micrographs
Movie frame alignment
Estimation of microscope CTF
Particle picking
Extraction
WARP
2D classification
Topaz train
Topaz extraction
2D classification
Combine & Remove duplicates
(cutoff distance 90Å)
Ab-initio reconstruction : C1
Heterogeneous refinement : C1
cryoSPARC
NUR : C1
Polishing
RELION
Ab-initio reconstruction : C1
Heterogeneous refinement : C1
selected
classes not selected
O
X
O
X
selected
NUR with per-particle defocus
(811,069 particles) : C1
RELION
Focused 3D classification
Local refinement
(299,537 particles) : C1
NUR with per-particle defocus
(1,280,178 particles) : C1
Heterogeneous refinement : C1
NUR with per-particle defocus
(776,754 particles) : C3
Symmetry expansion : C3
NUR with per-particle defocus : C3
Local refinement
(2,330,259 particles) : C1
cryoSPARC
e
RBD : TRI2-2
NTD
2.1
2.7
3.3
3.9
4.5
Å
f
RBD : TRI2-2
g
NTD

Supplementary Figure 4. CryoEM data collection and refinement of TRI2-2 bound to the BA.2.86 S glycoprotein trimer. (a-b) Representative electron micrograph (**a**) and 2D class averages (**b**) of SARS-CoV-2 BA.2.86 S in complex with TRI2-2. The scale bar represents 100nm (**a**) and 210Å (**b**). **(c)** Gold-standard Fourier shell correlation curves for the cryoEM reconstructions. The 0.143 cutoff is indicated with a gray dashed line. Black, gray, and blue curves correspond to the global, RBD, and NTD reconstructions, respectively. **(d)** Data processing flowchart. NUR: non-uniform refinement. Masks used for local refinement are shown in red. **(e-g)** CryoEM map of SARS-CoV-2 BA.2.86 S in complex with TRI2-2 (**e**), locally refined map of the BA.2.86 RBD in complex with TRI2-2 (**f**), and locally refined map of the BA.2.86 NTD (**g**) colored by local resolution as determined using cryoSPARC.

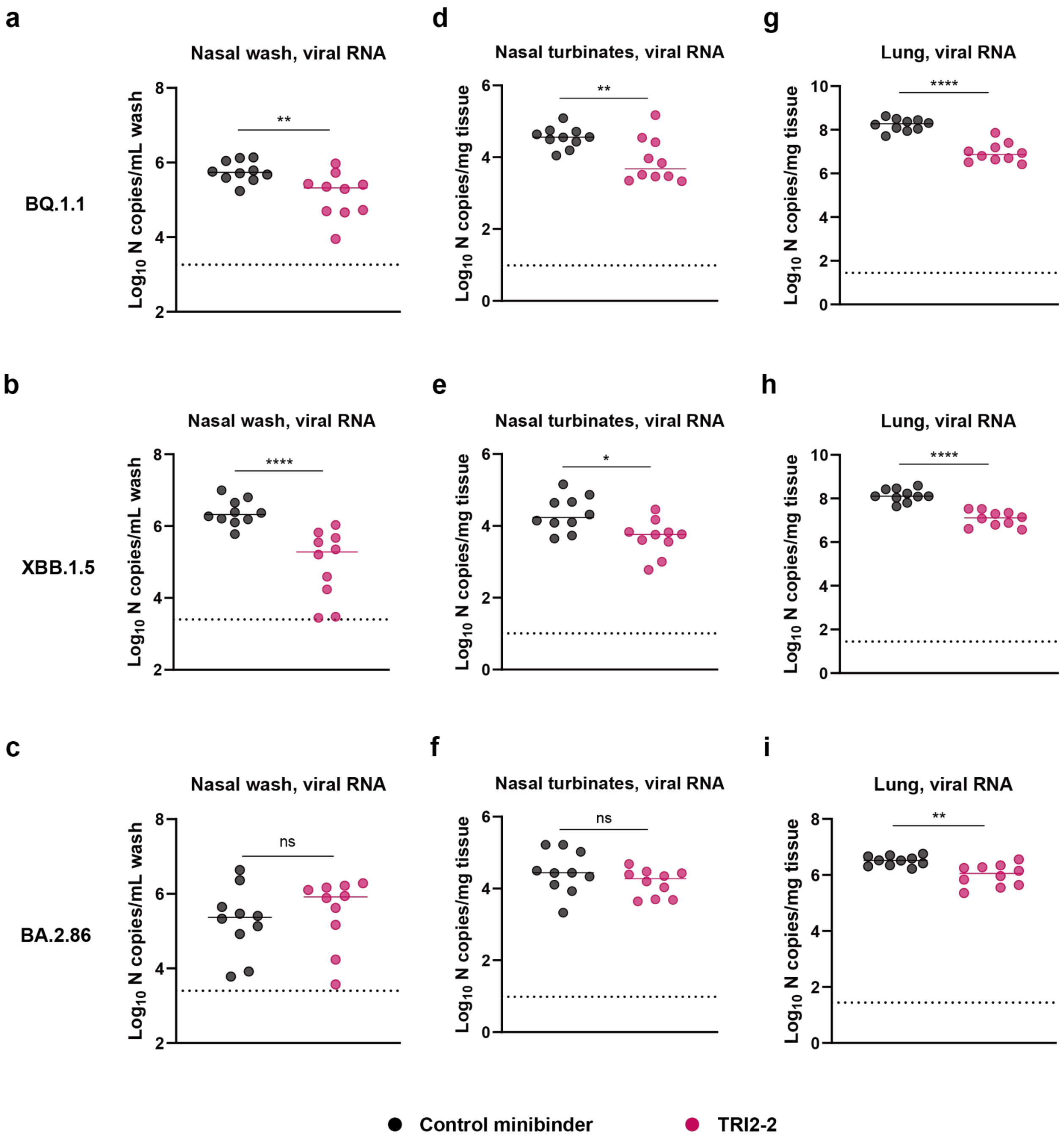

Supplementary Figure 5. Quantification of viral RNA loads. (a-c) Genomic viral RNA levels in nasal washes for mice challenged with BQ.1.1 (**a**), XBB.1.5 (**b**), or BA.2.86 (**c**). **(d-f)** Genomic viral RNA levels in nasal turbinates for mice challenged with BQ.1.1 (**d**), XBB.1.5 (**e**), or BA.2.86 (**f**). **(g-i)** Genomic viral RNA levels in lungs for mice challenged with BQ.1.1 (**g**), or XBB.1.5 (**h**), or BA.2.86 (**i**) (solid lines indicate median values; dotted lines indicate limit of detection of assay, n = 10 mice per group per virus challenge, two experiments; Two-tailed Mann-Whitney test between control and TRI2-2 treatment; ns, not significant; *$p < 0.05$, **$p < 0.01$, ***$p < 0.001$, ****$p < 0.0001$, $p=0.0089$ (a), $p<0.0001$ (b), $p=0.4359$ (c), $p=0.0089$ (d), $p=0.0185$ (e), $p=0.2176$ (f), $p<0.0001$ (g), $p<0.0001$ (h), $p=0.0015$ (i).

Supplementary Table 1. Representative TRI2-2 binding kinetics and avidities (apparent affinities denoted $K_{D,app}$) to SARS-CoV-2 variant RBDs obtained by biolayer interferometry. Values shown here are calculated from the curve fit from Supplementary Figure 1.

	$K_{D,app}$ (M)	$K_{D,app}$ error	k_{on} (1/Ms)	k_{on} error	k_{off} (1/s)	k_{off} error
**D614G**	1.21E-12	N/A	2.86E+05	2.42E+02	3.46E-07	N/A
**Delta**	1.14E-12	N/A	2.59E+05	4.17E+02	2.95E-07	N/A
**BA.1**	9.28E-10	6.07E-12	2.84E+05	5.09E+02	2.64E-04	1.66E-06
**BA.2**	4.93E-10	5.82E-12	2.97E+05	5.47E+02	1.47E-04	1.71E-06
**BA.2.12.1**	8.51E-10	8.90E-12	1.37E+05	2.93E+02	1.17E-04	1.20E-06
**BA.2.75.2**	5.42E-10	6.40E-12	1.65E+05	2.67E+02	8.93E-05	1.05E-06
**BA.5**	3.06E-11	4.60E-12	3.25E+05	4.97E+02	9.92E-06	1.49E-06
**BQ.1.1**	2.10E-10	5.49E-12	1.60E+05	2.23E+02	3.36E-05	8.78E-07
**XBB.1.5**	6.58E-10	7.02E-12	1.60E+05	2.78E+02	1.05E-04	1.11E-06
**BA.2.86**	3.88E-10	5.79E-12	3.39E+05	6.69E+02	1.31E-04	1.95E-06
**JN.1**	6.19E-09	6.11E-11	2.82E+05	2.42E+03	1.75E-03	8.53E-06
**KP.3**	N/A	N/A	N/A	N/A	N/A	N/A

Supplementary Table 2. CryoEM data collection and refinement statistics.

	**SARS-CoV-2 S BA.2.86 in complex with TRI2-2 minibinder**	**SARS-CoV-2 BA.2.86 RBD in complex with TRI2-2 minibinder**	**SARS-CoV-2 BA.2.86 NTD**
**Data collection and processing**	EMD-45972<br>PDB 9CWR	EMD-45969<br>PDB 9CWP	EMD-45971<br>PDB 9CWQ
**Magnification**	**105,000**	**105,000**	**105,000**
**Voltage (kV)**	**300**	**300**	**300**
**Electron exposure ($e^{-}/Å^2$)**	**53.25**	**53.25**	**53.25**
**Defocus range (μm)**	**-0.8 - -1.8**	**-0.8 - -1.8**	**-0.8 - -1.8**
**Pixel size (Å)**	**0.829**	**0.829**	**0.829**
**Symmetry imposed**	**C1**	**C1**	**C1**
**Final particle images (no.)**	**811,069**	**299,537**	**2,330,259**
**Map resolution (Å)**	**2.4**	**3.2**	**2.8**
**FSC threshold**	**0.143**	**0.143**	**0.143**
**Map sharpening B factor ($Å^2$)**	**-76.2**	**-117.8**	**-91.7**
**Validation**			
**MolProbity score**	**1.04**	**0.82**	**1.64**
**Clashscore**	**2.21**	**0.79**	**2.33**
**Poor rotamers (%)**	**0.88**	**0.54**	**2.69**
**Ramachandran plot**			
**Favored (%)**	**97.80**	**97.68**	**95.73**
**Allowed (%)**	**2.20**	**2.32**	**4.27**
**Disallowed (%)**	**0.00**	**0.00**	**0.00**
