# Supplementary material for: The computationally designed TRI2-2 miniprotein inhibitor protects against multiple SARS-CoV-2 Omicron variants
Source: Commun Biol. 2026 Jan 10;9:224. doi: 10.1038/s42003-025-09499-2 (PMC12901202; doi:10.1038/s42003-025-09499-2)
Supplement: Supplementary file 2 — Description of Additional Supplementary Files [file 42003_2025_9499_MOESM2_ESM.pdf]

Description of Additional Supplementary File

File name: Supplementary_Data_1
Description: The source data for all graphs in the paper
