# Supplementary material for: The computationally designed TRI2-2 miniprotein inhibitor protects against multiple SARS-CoV-2 Omicron variants
Source: Commun Biol. 2026 Jan 10;9:224. doi: 10.1038/s42003-025-09499-2 (PMC12901202; doi:10.1038/s42003-025-09499-2)
Supplement: Supplementary file 4 — Reporting Summary [file 42003_2025_9499_MOESM4_ESM.pdf]

nature portfolio

Corresponding author(s): David Veesler

Last updated by author(s): Dec 12, 2025

Reporting Summary

Nature Portfolio wishes to improve the reproducibility of the work that we publish. This form provides structure for consistency and transparency in reporting. For further information on Nature Portfolio policies, see our Editorial Policies and the Editorial Policy Checklist.

Statistics

For all statistical analyses, confirm that the following items are present in the figure legend, table legend, main text, or Methods section.

n/a	Confirmed	
☐	☒	The exact sample size (*n*) for each experimental group/condition, given as a discrete number and unit of measurement
☐	☒	A statement on whether measurements were taken from distinct samples or whether the same sample was measured repeatedly
☐	☒	The statistical test(s) used AND whether they are one- or two-sided<br>*Only common tests should be described solely by name; describe more complex techniques in the Methods section.*
☐	☒	A description of all covariates tested
☐	☒	A description of any assumptions or corrections, such as tests of normality and adjustment for multiple comparisons
☐	☒	A full description of the statistical parameters including central tendency (e.g. means) or other basic estimates (e.g. regression coefficient) AND variation (e.g. standard deviation) or associated estimates of uncertainty (e.g. confidence intervals)
☐	☒	For null hypothesis testing, the test statistic (e.g. *F*, *t*, *r*) with confidence intervals, effect sizes, degrees of freedom and *P* value noted<br>*Give P values as exact values whenever suitable.*
☒	☐	For Bayesian analysis, information on the choice of priors and Markov chain Monte Carlo settings
☒	☐	For hierarchical and complex designs, identification of the appropriate level for tests and full reporting of outcomes
☒	☐	Estimates of effect sizes (e.g. Cohen's *d*, Pearson's *r*), indicating how they were calculated

Our web collection on statistics for biologists contains articles on many of the points above.

Software and code

Policy information about availability of computer code

Data collection	serialEM
Data analysis	Coot-v0.9.8.1, cryoSPARC, GraphPad Prism-v10.1.1, Relion, Rosetta, UCSF Chimera-v1.16, UCSF ChimeraX-v1.8, WARP-v1.10, Phenix-v1.21, Molprobity, Privateer

For manuscripts utilizing custom algorithms or software that are central to the research but not yet described in published literature, software must be made available to editors and reviewers. We strongly encourage code deposition in a community repository (e.g. GitHub). See the Nature Portfolio guidelines for submitting code & software for further information.

Data

Policy information about availability of data

All manuscripts must include a data availability statement. This statement should provide the following information, where applicable:

- Accession codes, unique identifiers, or web links for publicly available datasets
- A description of any restrictions on data availability
- For clinical datasets or third party data, please ensure that the statement adheres to our policy

The sharpened and unsharpened cryoEM reconstructions and atomic models of SARS-CoV-2 BA.2.86 S in complex with TRI2-2 minibinder, SARS-CoV-2 BA.2.86 RBD in complex with TRI2-2 minibinder, and SARS-CoV-2 BA.2.86 NTD have been deposited in the Electron Microscopy Data Bank and the Protein Data Bank with accession codes EMD-45972 and PDB 9CWR (SARS-CoV-2 BA.2.86 S in complex with TRI2-2 minibinder), EMD-45969 and PDB 9CWP (SARS-CoV-2 BA.2.86 RBD in

complex with TRI2-2 minibinder), and EMD-45971 and PDB 9CWQ (SARS-CoV-2 BA.2.86 NTD). The source data underlying the figures can be found in the Supplementary Data 1 file. Other data will be available from the corresponding author upon request.

Research involving human participants, their data, or biological material

Policy information about studies with human participants or human data. See also policy information about sex, gender (identity/presentation), and sexual orientation and race, ethnicity and racism.

Reporting on sex and gender	N/A
Reporting on race, ethnicity, or other socially relevant groupings	N/A
Population characteristics	N/A
Recruitment	N/A
Ethics oversight	N/A

Note that full information on the approval of the study protocol must also be provided in the manuscript.

Field-specific reporting

Please select the one below that is the best fit for your research. If you are not sure, read the appropriate sections before making your selection.

☒ Life sciences ☐ Behavioural & social sciences ☐ Ecological, evolutionary & environmental sciences

For a reference copy of the document with all sections, see nature.com/documents/nr-reporting-summary-flat.pdf

Life sciences study design

All studies must disclose on these points even when the disclosure is negative.

Sample size	No sample-size calculations were performed to power each study. Instead, sample sizes were determined based on prior in vivo virus challenge experiments.
Data exclusions	No data were excluded from the analysis.
Replication	Experimental assays were performed at least two to four independent replicates. Each replicates was performed with 1~3 technical replicates. All replicates were reproducible.
Randomization	Randomization and blinding were not applicable due to pre-defined housing conditions.
Blinding	In vivo studies were not blinded, and mice were randomly assigned to treatment groups.

Reporting for specific materials, systems and methods

We require information from authors about some types of materials, experimental systems and methods used in many studies. Here, indicate whether each material, system or method listed is relevant to your study. If you are not sure if a list item applies to your research, read the appropriate section before selecting a response.

Materials & experimental systems

n/a	Involved in the study	
☒	☐	Antibodies
☐	☒	Eukaryotic cell lines
☒	☐	Palaeontology and archaeology
☐	☒	Animals and other organisms
☒	☐	Clinical data
☒	☐	Dual use research of concern
☒	☐	Plants

Methods

n/a	Involved in the study	
☒	☐	ChIP-seq
☒	☐	Flow cytometry
☒	☐	MRI-based neuroimaging

Eukaryotic cell lines

Policy information about cell lines and Sex and Gender in Research

Cell line source(s)	HEK293T (ATCC, CRL-11268), Vero E6-TMPRSS2-GFP11, BHK-21-GFP1–10, HEK293T cells with stable human ACE2 expression, Expi293
Authentication	None of the cell lines used were authenticated.
Mycoplasma contamination	Cell lines were not tested for mycoplasma contamination.
Commonly misidentified lines (See ICLAC register)	No commonly misidentified cell lines were used in the study.

Animals and other research organisms

Policy information about studies involving animals; ARRIVE guidelines recommended for reporting animal research, and Sex and Gender in Research

Laboratory animals	Heterozygous K18-hACE2 C57BL/6J mice (strain: 2B6.Cg-Tg(K18-ACE2)2Prlmn/J) were obtained from The Jackson Laboratory.
Wild animals	This study did not involve wild animals.
Reporting on sex	8-week-old female K18-hACE2 mice were used for the study.
Field-collected samples	No field collected samples were used in this study.
Ethics oversight	Mouse experiments Animal studies were carried out in accordance with the recommendations in the Guide for the Care and Use of Laboratory Animals of the National Institutes of Health. The protocols were approved by the Institutional Animal Care and Use Committee at the Washington University School of Medicine (assurance number A3381-01). Virus inoculations were performed under anesthesia that was induced and maintained with ketamine hydrochloride and xylazine, and all efforts were made to minimize animal suffering.

Note that full information on the approval of the study protocol must also be provided in the manuscript.

Plants

Seed stocks	N/A
Novel plant genotypes	N/A
Authentication	N/A
